# Considering Strain Variation and Non-Type Strains for Yeast Metabolic Engineering Applications

**DOI:** 10.3390/life12040510

**Published:** 2022-03-30

**Authors:** Xiunan Yi, Hal S. Alper

**Affiliations:** 1 Interdisciplinary Life Sciences, The University of Texas at Austin, Austin, TX 78712, USA; xiunan.yi@utexas.edu; 2 McKetta Department of Chemical Engineering, The University of Texas at Austin, Austin, TX 78712, USA

**Keywords:** metabolic engineering, yeast cell factory, non-type strains, strain variations, strain selection

## Abstract

A variety of yeast species have been considered ideal hosts for metabolic engineering to produce value-added chemicals, including the model organism *Saccharomyces cerevisiae*, as well as non-conventional yeasts including *Yarrowia lipolytica*, *Kluyveromyces marxianus*, and *Pichia pastoris*. However, the metabolic capacity of these microbes is not simply dictated or implied by genus or species alone. Within the same species, yeast strains can display distinct variations in their phenotypes and metabolism, which affect the performance of introduced pathways and the production of interesting compounds. Moreover, it is unclear how this metabolic potential corresponds to function upon rewiring these organisms. These reports thus point out a new consideration for successful metabolic engineering, specifically: what are the best strains to utilize and how does one achieve effective metabolic engineering? Understanding such questions will accelerate the host selection and optimization process for generating yeast cell factories. In this review, we survey recent advances in studying yeast strain variations and utilizing non-type strains in pathway production and metabolic engineering applications. Additionally, we highlight the importance of employing portable methods for metabolic rewiring to best access this metabolic diversity. Finally, we conclude by highlighting the importance of considering strain diversity in metabolic engineering applications.

## 1. Introduction

Microbial cell factories provide a renewable means to produce value-added compounds [1,2,3]. In contrast to using fossil fuels in chemical synthesis, microbes can utilize renewable bio-resources and alternative substrates, such as agricultural and environmental waste, to build carbon-based molecules [4]. This shift to sustainable and renewable production is possible due to advances in metabolic engineering. To this end, a significant number of bio-derived chemicals have been demonstrated using microbial cell factories including industrial precursors [5], fatty acid derivatives [6], nutrition and health supplements [7], pharmaceuticals [8], and other plant-derived natural products [9].

Across potential microbes of interest, yeasts have unique advantages as eukaryotic microbial cell factories [10]. As points of evidence, physiological and metabolic differences across yeast hosts, including model yeast *Saccharomyces cerevisiae* and non-conventional yeasts *Yarrowia lipolytica*, *Kluyveromyces marxianus*, and *Pichia pastoris*, have enabled a high-level production of various polyketides [11,12], terpenes [13,14], and alkaloids [15,16]. Existing P450-related enzymes and secondary metabolite intermediates in yeasts make them uniquely suitable for the production of plant-based products [9]. In other cases, oleaginous yeast *Y. lipolytica* and *Rhodosporidium toruloides* have also been optimized to produce fatty acids and their derivatives [17,18]. Across these hosts, exquisite fermentation condition tolerization (esp. in species like *Y. lipolytica* and *K. marxianus*) [19,20] are favorable for industrial productions. A number of thorough reviews have been published on the topic of yeast cell factories [21,22,23] and metabolic engineering of non-conventional yeasts [24,25,26].

Even within a specific yeast genus and species, there exists a wide range of strain variation and metabolic potential. Recent studies have highlighted the importance of this variation for successful metabolic engineering. For example, the production of triacetic acid lactone was seen to vary by up to 63-fold across 13 industrial *S. cerevisiae* strains [27]. *S. cerevisiae* CEN.PK was reported to be a better host for *p*-coumaric acid production compared with *S. cerevisiae* S288C [28]. Engineered *S. cerevisiae* Sigma strains produce more itaconic acid and 2,3-butanediol than engineered CEN.PK and BY4741 strains in the same condition [29,30]. These examples point toward an important facet of metabolic engineering: how to identify the best starting strain.

With such production variation abound, the ability to rapidly prototype metabolism through classic and new genetic tools is enabling a broader survey of metabolic potential. Plasmid-based overexpression is generally portable across different strains within the same species [27,28,29,30]. In addition, advances in genetic manipulation through the use of clustered regularly interspaced short palindromic repeats (CRISPR) and RNA interference (RNAi) contribute to the development of new yeast cell factories [29,31]. Specifically, CRISPR techniques not only accelerate genome-scale engineering in yeasts such as *S. cerevisiae* [32] but also establish standardized approaches for rapid metabolic engineering in non-conventional yeasts [33]. These advances help bypass the previously limited set of genetic tools for these hosts. Certainly, the topic of CRISPR-mediated genome and metabolic engineering in yeasts has been reviewed extensively in the literature [34,35,36,37].

**Figure 1:**
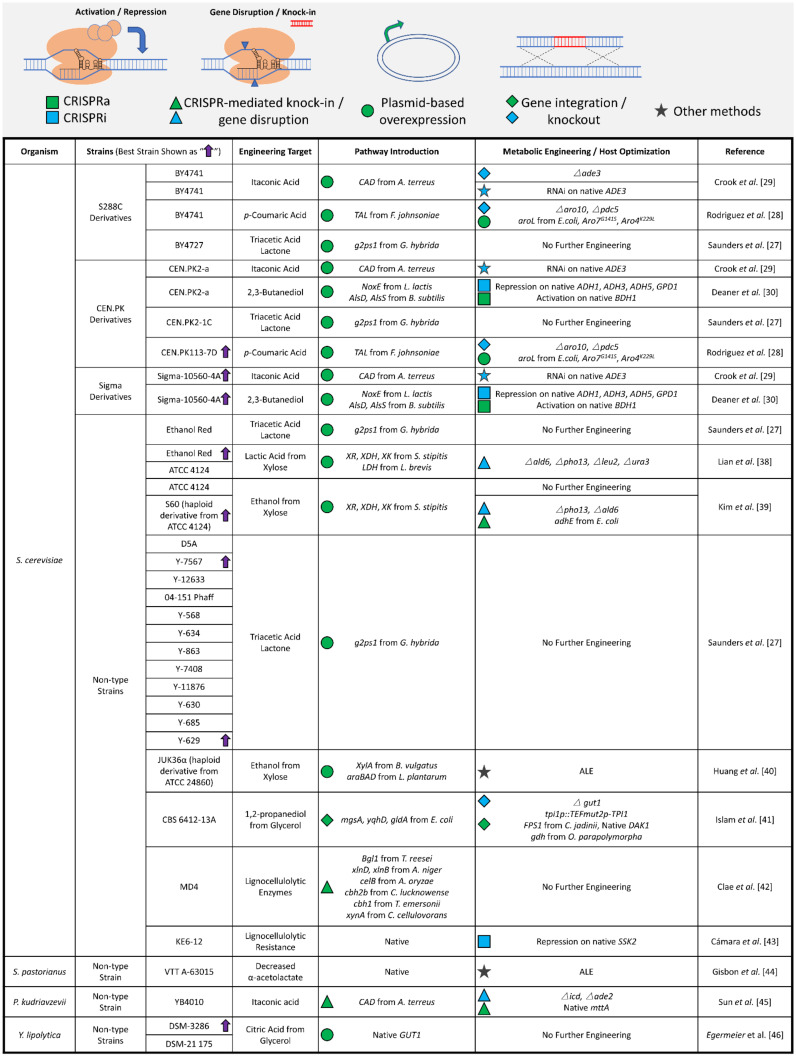
Summary of metabolic engineering across different yeast strains. A graphic table summarizes the metabolic engineering efforts across both common laboratory yeast strains and non-type yeast strains. The varied approaches to achieving metabolic rewiring in these strains are illustrated (Green □, CRISPR activation; Blue □, CRISPR interference; Green △, CRISPR-mediated gene disruption; Blue △, CRISPR-mediated knock-in; Green ◯, plasmid-based overexpression; Green ◇, gene integration; Blue ◇, gene knockout; ☆, other methods) with corresponding gene targets. Purple ↑: the best producing strains (when compared with other strains) reported in the studies [27,28,29,30,38,39,40,41,42,43,44,45,46].

It will take the combination of diverse starting strains with new genome editing techniques to make truly effective microbial cell factories of the future. In this review, we specifically highlight recent advances that survey yeast metabolic potential, assess non-model and non-type strains for metabolic engineering, and employ portable methods for metabolic rewiring, all of which are summarized in Figure 1. Through this review, we provide a perspective on how to establish more efficient microbial biotransformation.

## 2. Surveying Innate Metabolic Production and Rewiring Potential across Strains

Among the possible choice of hosts, “type strains” are of great interest for research and development as they come with the advantages of sequenced genomes, characterized metabolism, well-studied genetic and enzymatic regulations, and existing selection markers [47,48]. It is thus not surprising that these types of strains play a major role in both fundamental molecular biology studies and in applied studies to create microbial cell factories via metabolic engineering. However, metabolic phenotypes and general physiology differ across strain backgrounds, even within the same species. These differences have been noted in literature in examples such as growth physiology and stress sensitivity of *S. cerevisiae* [49,50], the citric acid production from *Y. lipolytica* [51], and the heat tolerance of *K. marxianus* [52]. These known variations can influence not just innate production, but also respond to metabolic engineering strategies in surprising ways. In this section, we summarize various studies that explore strain variation as an integral part of the metabolic engineering design cycle.

While reconstituting a synthetic RNAi system [53] in *S. cerevisiae*, Crook et al. reported on strain variations in the basal production of itaconic acid, as well as on the response to RNAi-based reprogramming [29]. Specifically, the authors introduced optimized RNAi machinery into three laboratory *S. cerevisiae* strains: BY4741, CEN.PK, and Sigma and validated function. Next, the authors selected heterologous itaconic acid production [54] aided by the expression of cis-aconitic acid decarboxylase from *Aspergillus terreus*, combined with an RNAi system targeting *ADE3*, a previously reported target [55]. In doing so, Crook et al. observed differences in the fold change of itaconic acid titer across all three strains with the Sigma strain producing the highest itaconic acid titer. This study not only showed that the same heterologous RNAi machinery can be functionally deployed across stains but also demonstrated that the response to this rewiring can be distinct.

In another study, Saunders et al. aimed to explore the difference in triacetic acid lactone (TAL) production across 13 laboratory and industrial *S. cerevisiae* strains with varied genetic backgrounds [27] using a heterologous expression approach. In this system, TAL can be synthesized from acetyl-CoA and malonyl-CoA through the expression of a 2-pyrone synthase (*g2ps1*). The TAL titer and yield across strains were compared using different carbon sources including glucose, ethanol, acetate, and glycerol. The authors found up to a 63-fold difference in TAL titer across strains when the *g2ps1* was initially expressed by the *ADH2* promoter using glucose as the carbon source. Switching this expression to the *TEF1* promoter resulted in more consistent TAL production, but still exhibited strain-specific variation. This variation was also significantly affected by the carbon source. Strain Y-629 was the best TAL producer in ethanol and acetate, whereas strain Y-7567 produced the most TAL in glucose. Ultimately, this work selected strain Y-629 in an ethanol fed-batch fermentation to achieve 5.2 g/L of TAL. This study points out the importance of strain selection in creating yeast cell factories as laboratory strains including BY4727 and CEN.PK, which were outcompeted by non-type strains in all carbon sources. In addition, the results indicated that the nexus between strain background and environmental conditions (in this case, carbon sources) are difficult to predict without experimental validation.

In another study, Rodriguez et al. focused on the transcriptomic and metabolomic changes between two laboratory *S. cerevisiae* strains CEN.PK and BY4741 (an S288C derivative) after introducing a *p*-coumaric acid (p-CA) pathway and further optimizing for its production [28]. For each strain, two genetically modified p-CA producers were generated comprising a “low-producer”, with only a tyrosine ammonia-lyase from *Flavobacterium johnsoniae* to convert tyrosine into p-CA, and a “high-producer” that additionally contained the overexpression of *aroL* from *Escherichia coli*, the feedback inhibition-resistant *ARO7^G141S^* and *ARO4^K229L^* from *S. cerevisiae*, and the deletion of *aro10* and *pdc5*. Both CEN.PK producers had a higher p-CA titer than the corresponding S288C producers. A pairwise transcriptomic comparison using the “high-producers” indicated that the CEN.PK strain had a far less perturbed transcriptome than the S288C strain. Notably, genes involved in amino acid and protein synthesis were downregulated in the S288C “high-producer”, thus impacting its production capability. Thus, Rodriguez et al. claimed that CEN.PK was more suited for p-CA production and other aromatic compounds over S288C. This study provided evidence that strain variation matters in p-CA production and likewise provided an analysis of metabolic rewiring/transcriptional response, thus raising the prospect that the same metabolic engineering strategy may not function in different strains.

In a final example of evaluating multiple strains, Deaner et al. applied a sgRNA array for the multiplex rewiring of gene expression using CRISPR [30]. Specifically, the use of tRNA-sgRNA-tRNA (TST) operons under the control of a strong Pol II promoter enabled a transferrable, multiplex rewiring of cells. With this approach, Deaner et al. constructed a sgRNA array to achieve the repression of *adh1*, *adh3*, *adh5*, and *gpd1*, and the activation of *BDH1* simultaneously in an effort to optimize the 2,3-butanediol (BDO) production in two laboratory *S. cerevisiae* strains, CEN.PK and Sigma. In both strains, the expression of the sgRNA array resulted in a 2-fold increase in BDO titer. However, Sigma strain produced substantially more BDO than CEN.PK both before and after the optimization (more than a 3-fold difference). This study demonstrated both the difference in innate metabolism as well as the portability of CRISPR technologies for rewiring hosts.

These highlighted studies demonstrate the importance of strain variation in the overall design cycle. However, a more comprehensive strain selection process is not commonly used in the field wherein typical approaches rely upon a small handful of laboratory strains. Yet, the metabolic potential and even the rewiring strategy can be influenced by the strain. In some of these cases, the cellular response to RNAi and CRISPR rewiring and effectiveness varied. Thus, surveys such as these should be conducted throughout metabolic engineering endeavors. It is important to see that strains with the highest basal production may not yield the highest rewired production [56].

## 3. Successes in the Metabolic Engineering and Optimization of Non-Type Strains

While the examples in the section above demonstrate the utility of screening strain variation during the design cycle, sometimes even the singular selection of a non-model or non-type strain can lead to improved outcomes. Specifically, industrial strains generally feature fast growth, high tolerance to fermentation conditions, the capability of metabolizing multiple carbon sources, and/or the capacity to produce desired compounds [57]. However, many of these purely industrial hosts are challenging to engineer genetically owing to their polyploid nature, the requirement of dominant selection markers, and/or unknown genomic information. In this section, we highlight successful metabolic engineering efforts that have relied upon the use of non-type strains.

*Saccharomyces*-related strains. Kim et al. metabolically engineered a triploid industrial *S. cerevisiae* strain (ATCC 4124) [39] for the ability to utilize xylose. This strain was able to ferment glucose rapidly with high ethanol productivity compared with common laboratory strains and additionally exhibited high tolerance to heat and hydrolysate inhibitors, thus making it a good candidate for cellulosic ethanol production. To accomplish this engineering, one copy of a xylose utilization pathway (*XR*, *XDH*, and *XK*) from *Scheffersomyce stipitis* was integrated into the ATCC 4124 genome. To further optimize the strain and enable further engineering, Kim et al. isolated a stable haploid derivative, 4124-S60, but the resulting strain showed decreased ethanol production. After deleting *pho13* and *ald6*, the final strain exhibited a 20% increased ethanol titer from hydrolysates compared with the engineered laboratory strains. This study provided an example of how an improved strain can be obtained from a polyploid industrial strain through tedious genetics and engineering.

Absent of genetic tools, the approach of adaptive laboratory evolution (ALE) is commonly used for industrial strains especially for brewing and sake yeasts to improve strain performance and enrich beverage taste. Gibson et al. conducted ALE on a Frohberg-type lager yeast *Saccharomyces pastorianus* VTT A-63015 (A15) to reduce α-acetolactate production, a precursor of diacetyl (2,3-butanedione) that contributes to off-flavors in beer [44]. To optimize the strain, A15 was exposed to ethyl methanesulfonate and then screened in media containing chlorsulfuron, a compound that specifically targets acetohydroxy acid synthase. After 150 generations, the evolved population exhibited a 4-fold increase in viability when exposed to chlorsulfuron and a resulting significant decrease in diacetyl and 2,3 pentanedione production during a 4-day fermentation. Owing to a lack of genome sequence and the multitude of mutations incurred, the exact mechanism behind decreased α-acetolactate production remains unclear. Nevertheless, ALE is a powerful tool that can be applied to other non-type strains.

Similarly, Huang et al. generated a hydrolysate-cofermenting *S. cerevisiae* strain from a haploid strain JUK36α, which was derived from ATCC 24,860 [40]. By introducing randomly assembled gene expression cassettes for xylose-metabolizing into the genome of JUK36α, strain diversity could be screened on xylose and arabinose plates. The best isolate, 36aS1, was then cultivated in pentose media containing acetic acid for ALE. After 10 passages, strains were screened on plates containing industrial hydrolysate. One of the isolates (specifically, 36aS1.10.4) was able to produce ethanol with increased titer by consuming both glucose and xylose. Industrial wheat straw hydrolysate fermentation using 36aS1.10.4 resulted in an ethanol titer of 54.11 g/L with maximal specific rates at 1.38 g/g DW/h. This study combined haploid engineering using ALE in non-type strains to establish stable pentose metabolizing.

Recently advanced CRISPR techniques provide a simpler solution to engineering non-type strains. Lian et al. developed a CRISPR/Cas9 genome editing system applicable to industrial yeast strains [38]. To overcome the major hurdle of decreased editing efficiency in polyploid strains that contain multiple copies of chromosomes, Lian et al. increased the copy numbers of sgRNA-containing plasmids. The editing efficiency of this optimized CRISPR system was demonstrated in two industrial *S. cerevisiae* strains: Ethanol Red (diploid) and ATCC 4124 (triploid, mentioned above). For Ethanol Red, four selected auxotrophic marker genes were deleted at 100% efficiency in a single step, while for ATCC 4124, three genes (*his3*, *leu2*, and *ura3*) were disrupted simultaneously also at 100% efficiency. Using this CRISPR approach, the authors engineered both Ethanol Red and ATCC 4124 by disrupting *ald6*, *pho13*, *leu2*, and *ura3* in a single step to improve the xylose utilization and provide two auxotrophic markers to introduce both the xylose utilization pathway (mentioned above) from *S. stipitis* and LDH from *Lactobacillus brevis* into the edited strains. The resulting strains were able to produce lactate from xylose, providing a convincing case that CRISPR-based genetic manipulation can be applied to non-type strains to generate optimized yeast cell factories. However, it is also important to note that the CRISPR editing was not able to delete all four auxotrophic markers in ATCC 4124 and the exact reason was unclear.

Beyond gene disruption, CRISPR-mediated knock-in techniques have been applied to non-type strains. Islam et al. modularly engineered a haploid glycerol metabolizing *S. cerevisiae* strain CBS 6412-13A to produce 1,2-propanediol (PDO) [41]. In this effort, the PDO synthesis pathway (*mgsA*, *yqhD*, and *gldA*) from *E. coli* and the overexpression of *FPS1* from *Cyberlindnera jadinii* was introduced into CBS 6412-13A as a first module. Subsequently, the native FAD-dependent glycerol catabolic pathway was replaced by a second module including the overexpression of *gdh* from *Ogataea parapolymorpha* and endogenous *DAK1* along with the deletion of endogenous *gut1*. The genomic integration of *mgsA* and *gldA* in the strain with both modules was achieved by CRISPR-mediated knock-in techniques. Finally, a third module was used to attenuate native *TPI1* expression. Islam et al. found that the introduction of second and third modules improved the PDO titer by 6-fold in nitrogen-limited media. The final strain produced 4.3 g/L PDO in a bioreactor, which is the highest PDO titer using engineered *S. cerevisiae* fermentation. The efficiency of CRISPR knock-in relies on the efficiency of homologous recombination in the organism, as well as an understanding of the genome sequence. While *S. cerevisiae* is known for its superior homologous recombination mechanism [58], the general applicability of CRISPR knock-in has not been fully surveyed in non-type strains.

CRISPR-mediated knock-in approaches have also enabled the direct production of ethanol from multiple lignocellulosic substrates. Claes et al. created a second-generation industrial *S. cerevisiae* strain, MD4, that simultaneously expresses and secretes seven lignocellulolytic enzymes [42]. Similar CRISPR techniques were used to sequentially integrate seven heterologous genes encoding for lignocellulolytic enzymes. The resulting strain produced about 94.5 filter paper units of lignocellulolytic enzymes per gram of cell dry weight while also exhibiting high utilization of both glucose and xylose. While promising, this work highlights some of the challenges with non-type strains. Specifically, the integration appeared unstable as the copy number of the first integrated gene decreased with subsequent integration events owing partially to the polyploidy of the strain (largely tetraploid, with some pentaploid chromosomes). This effort raised concerns about the CRISPR/Cas9-related off-target effect and genome instability [59] in the polyploid non-type strains. Specifically, it can be difficult to evaluate the off-target effect in non-type strains when overall genome sequences and general genetic information is unknown.

In a different application, Cámara et al. optimized and applied a CRISPR activation- and interference (CRISPRa/i)-based approach to control gene expression in the polyploid industrial *S. cerevisiae* strain KE6-12 [43]. The efficiency of different CRISPR effectors was evaluated by targeting the promoter region of mRuby2 in KE6-12. The authors found that the best targeting sites for activation (with dCas9-VP64 or dCas9-VPR) or repression (with dCas9 or dCas9-Mxi) for the same gene were different. Given the promise of KE6-12 for lignocellulosic hydrolysate utilization, Cámara et al. used this approach to target improved fermentation capabilities. Specifically, the authors found that the activation of *SSK2* significantly reduced the generation time and improved biomass yield when cultivated in the lignocellulosic hydrolysate. However, activation of three other previously reported gene targets did not show any significant improvement in the inhibitor tolerance. Cámara et al. suggested that unknown sequence variability may decrease the targeting efficiency given the fact that KE6-12 is a polyploid strain with an unknown genomic sequence. Although this particular CRISPRa/i strategy could only manipulate a single gene target at once, adapted strategies could be used to incorporate multiplexed sgRNA arrays. Moreover, it is still an open question whether the efficiency of targeting here would be improved with multiple sgRNAs targeting the same gene to help insulate sequence variation and increase the degree of actuation.

## 4. Non-Conventional Yeasts

Metabolic engineering examples in non-type strains is not limited to simply *Saccharomyces*. *Pichia kudriavzevii* is a non-conventional yeast with high organic acid tolerance and low pH conditions, highly desirable traits in downstream processing. Sun et al. utilized *P. kudriavzevii* to produce itaconic acid [45] via metabolic engineering strategies. It was noted that itaconic acid tolerance was generally much higher across 5 wild-type *P. kudriavzevii* strains compared with two laboratory *S. cerevisiae* strains. Subsequently, the cis-aconitic acid decarboxylase from *A. terreus* was introduced into *P. kudriavzevii* YB4010 along with overexpression of a native putative mitochondrial tricarboxylate transporter to double itaconic acid production. The genome integration of overexpression cassettes, as well as disruptions of isocitrate dehydrogenase, were achieved by CRISPR/Cas9-based editing. The resulting *P. kudriavzevii* strain improved the itaconic acid production by 4-fold compared with the starting strain, reaching 1.23 g/L in fed-batch fermentation. This study demonstrates the great potential of engineering the non-type and non-conventional host *P. kudriavzevii* with CRISPR techniques to build industrial cell factories for organic acid production.

Another non-conventional yeast, *Y. lipolytica*, has been increasingly viewed as an ideal host for yeast cell factories due to its native high flux towards lipid and tolerance to low pH and other fermentation conditions [60]. Egermeier et al. designed a method for rapid metabolic engineering for wild-type *Y. lipolytica* strains with unknown genetic backgrounds [46]. Using a Golden Gate assembly scheme, this method can efficiently generate overexpression cassettes or build CRISPR gene disruptions. To demonstrate the effectiveness of this method, glycerol kinase was overexpressed in two wild-type *Y. lipolytica* strains, DSM-3286 and DSM-21175. Both engineered strains exhibited an increased glycerol utilization rate but showed distinctive differences in citric acid production. The engineered DSM-3286 was able to produce more than 40 g/L of citric acid in low pH conditions, whereas the engineered DSM-21175 produced less than 10 g/L. This study once again showcases the variation that exists between strain backgrounds. To investigate CRISPR gene disruptions, two sgRNAs targeting *LEU2* were designed using the genome sequence of *Y. lipolytica* W29, and *leu2* was deleted in both DSM-3286 and DSM-21175 with an efficiency of up to 25%. Differences between the two strains (likely at the sequence level) were observed as one of the sgRNA was less efficient in DSM-21175 compared to its efficiency in DSM-3286. This study nevertheless offered a simplified strategy for metabolic engineering in non-type strains of *Y. lipolytica* using the existing expression system and CRISPR gene disruption method.

Non-type strains represent easy access to high tolerances, effective carbon utilization, and/or enhanced carbon flux towards valuable products and pathways. As seen above, studies have shown that non-type strains can be better overall hosts for specific metabolic engineering traits when compared with commonly used “type strains”. Limited genetic information, complex genome architecture/ploidy, and synthetic tool availability remain barriers to entry in this space. Fortunately, in many cases, the non-type strains share expression system architecture with the type strains and thus many of the gene overexpression and CRISPR toolsets can be transferrable. Certainly, further genetic and metabolomic studies on non-type strains will accelerate the metabolic engineering in those strains.

## 5. Conclusions

These studies (Figure 1) collectively illustrate some important considerations for metabolic engineering. Specifically, (1) strain variation must be considered to identify optimal performing cells, (2) basal production levels are not necessarily indicative of rewiring potential, and (3) a variety of generalizable tools are required to tackle strain engineering in non-conventional and non-type organisms. For the latter point, rapid metabolic engineering strategies featuring streamlined plug-and-play cloning [61,62,63] and CRISPR-based metabolism rewiring [30,64,65] have been developed and tested. Despite these advances, major challenges in non-type strains include polyploid chromosomes and unknown genetic information. Nevertheless, CRISPR-based rewiring approaches, including multiplexed activation, interference, gene disruption, and knock-in, have great potential. The effectiveness of CRISPR proteins and effectors [66,67,68], the expression of sgRNA cassettes, and off-target effects need to be tested further when considering non-type strains.

With the challenges of engineering aside, strain selection is an underappreciated aspect of metabolic engineering and is poised to become an important parameter in the future. In many of the examples outlined above, the highest production was only achievable upon selecting a non-standard strain. As much as the importance of strain variation is recognized, it remains difficult to predict the strain performance without knowing the genetic, transcriptomic, and metabolomic information of the strains. Moreover, the rewiring potential of a strain is perhaps a more important metric. In this regard, screening through a large number of non-type strains is required and thus necessitates high-throughput screening along with rapid and portable metabolism rewiring approaches.

Although still in an early stage, the studies reviewed here provide valuable insights into the importance of considering strain diversity in metabolic engineering applications. With ongoing studies using non-type strains, it is clear that this is a new and important direction to expand yeast cell factories.

## Data Availability

Not applicable.
